# Sodium–Glucose Cotransporter 2 Inhibitors to Decrease the Uric Acid Concentration—A Novel Mechanism of Action

**DOI:** 10.3390/jcdd10070268

**Published:** 2023-06-22

**Authors:** Anna Kochanowska, Przemysław Rusztyn, Karolina Szczerkowska, Stanisław Surma, Aleksandra Gąsecka, Miłosz J. Jaguszewski, Łukasz Szarpak, Krzysztof J. Filipiak

**Affiliations:** 1 1st Chair and Department of Cardiology, Medical University of Warsaw, 02-091 Warsaw, Poland; kochanowska.anka@gmail.com (A.K.); prusztyn@gmail.com (P.R.); karolina.szczerkowska1@gmail.com (K.S.); 2 Faculty of Medical Sciences in Katowice, Medical University of Silesia, 40-752 Katowice, Poland; surma.stanislaw96@gmail.com; 3 1st Department of Cardiology, Medical University of Gdansk, 80-210 Gdansk, Poland; mjaguszewski@gumed.edu.pl; 4 Institute of Outcomes Research, Maria Sklodowska-Curie Medical Academy, 03-411 Warsaw, Poland; lukasz.szarpak@uczelniamedyczna.com.pl; 5 Henry JN Taub Department of Emergency Medicine, Baylor College of Medicine, Houston, TX 77030, USA; 6 Institute of Clinical Science, Maria Sklodowska-Curie Medical Academy, 03-411 Warsaw, Poland; krzysztof.filipiak@uczelniamedyczna.com.pl; 7 Department of Hypertensiology, Angiology and Internal Medicine, University of Medical Sciences, 61-701 Poznan, Poland

**Keywords:** sodium–glucose cotransporter 2 inhibitors, SGLT2i, flozins, uric acid, gout

## Abstract

Sodium–glucose cotransporter 2 inhibitors (SGLT2is) are glucose-lowering agents whose positive impact on cardiovascular risk has been described extensively. Not only do they influence lipid profile, blood pressure, atherosclerosis risk, hemoglobin level, and insulin resistance, but they also reduce cardiovascular events, all-cause mortality, and hospitalization rates. Some of these effects may be due to their impact on serum uric acid (SUA) concentration. Findings from nine meta-analyses showed that, indeed, SGLT2is significantly reduce SUA. The data on the drug- and dose-dependency of this effect were inconclusive. Several factors alternating the beneficial effects of SGLT2is on SUA, such as glycated hemoglobin concentration (HbA1c), presence of diabetes, and baseline SUA level, were described. Even though there is a consensus that the lowering of SUA by SGLT2is might be due to the increased urinary excretion rate of uric acid (UEUA) rather than its altered metabolism, the exact mechanism remains unknown. The influence of SGLT2is on SUA may not only be used in gout treatment but may also be of huge importance in explaining the observed pleiotropic effects of SGLT2is.

## 1. Introduction

Sodium–glucose cotransporter 2 inhibitors (SGLT2is) are new glucose-lowering agents that are widely used in type 2 diabetes mellitus (T2DM) patients, as well as in nondiabetic patients, to improve their cardiovascular and metabolic outcomes. The positive impact on cardiovascular risk has been described extensively, together with kidney-protective effects, regardless of the presence of diabetes [1]. Other multiple promising effects of SGLT2is are being studied, in the hope that SGLT2is will be recognized as a holy grail of numerous diseases of affluence.

The considerably growing interest in uric acid (UA) is related to the dependence of UA levels in modern humans on lifestyle, diet, or environment [2]. Elevated serum uric acid (SUA) concentrations are the subject of much research due to their multifactorial effects on the body. The liver, intestines, and vascular endothelium mainly produce UA as the final product of an exogenous pool of purines. Additionally, UA is produced during the apoptosis of cells and the breakdown of nucleic acids, adenine, and guanine. This review evaluates the effect of SGLT2is on lowering SUA concentration, the mechanism of its reduction, and the factors influencing this effect. Additionally, it examines the role of UA as a risk factor for numerous diseases and provides current knowledge about the cardio–renal effects of SGLT2is.

## 2. Uric Acid—Multifactorial Effects on the Human Body

**Figure 1:**
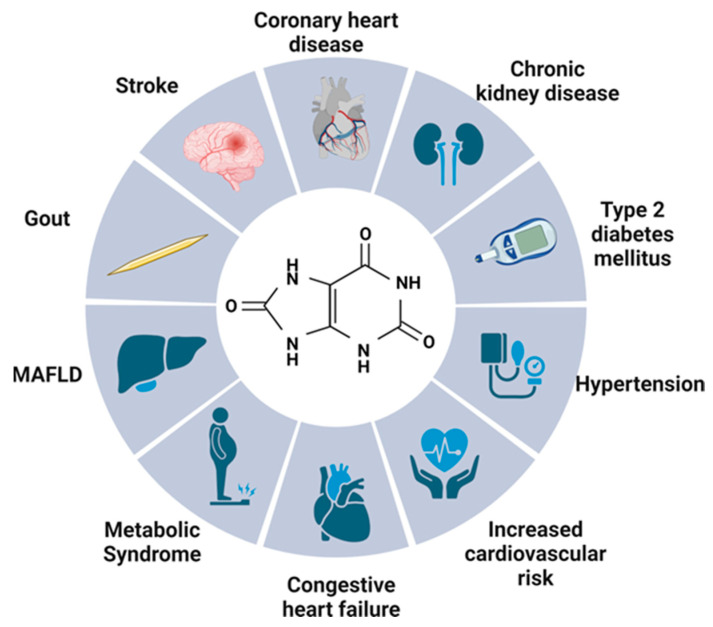
Role of increased level of uric acid in pathophysiology of numerous diseases. Abbreviations: MAFLD, metabolic dysfunction-associated fatty liver disease; Created with BioRender.com (accessed on 6 May 2023).

UA functions as a potent antioxidant, but also as a promoter of reactive oxygen species (ROS) and peroxynitrite [3], which contribute to the development of numerous diseases. The role of UA in the pathophysiology of numerous diseases is illustrated in Figure 1.

### 2.1. Gout

One of the best-documented effects of SUA is its correlation with gout. Gout was one of the earliest described disease entities that is closely related to high UA concentrations. One of the most prevalent types of inflammatory arthritis is linked to high levels of monosodium urate crystals present in tissues, which results in acute arthritis. In gout, SUA levels of <6 mg/dL are recommended, with the aim of preventing the creation of new crystals in the joints [4].

### 2.2. Kidneys

Roughly two-thirds of the UA load are removed by the kidneys, while the gastrointestinal tract is responsible for eliminating the remaining one-third. Therefore, a link between high SUA levels and kidney impairment is frequently a subject for consideration. Undoubtedly, there is a relationship between UA and the urinary system, including the mundane possibility of UA crystals precipitating and forming stones that cause nephrolithiasis. Research performed in the Chinese population revealed that hyperuricemia was linked to a more significant decrease in kidney function and a higher probability of developing kidney failure [5]. A greater decline in the estimated glomerular filtration rate (eGFR) was observed in patients with higher SUA, which can be considered a potential factor of chronic kidney disease (CKD) progression [6]. Similar conclusions were drawn by Ponticelli et al., where SUA-lowering agents have not produced evidence of improved kidney function [7]. However, the results are still controversial, and the correlation between lowering SUA and improved kidney function continues to be disputed.

### 2.3. Metabolic Syndrome

Lin et al. showed that SUA levels are positively associated with the risk factors of metabolic syndrome (MetS). High SUA levels were associated with obesity, body mass index (BMI), waist circumference (WC), and waist-to-hip ratio (WHR) [8]. An elevated insulin resistance index (HOMA-IR) in patients with hyperuricemia was also noted. Another study conducted in the Chinese population is also in agreement about the association between SUA and WC or triglycerides [9].

### 2.4. Type 2 Diabetes Mellitus

According to a study on the correlation between hyperuricemia and T2DM risk, patients with elevated SUA are at a greater risk of T2DM [10,11]. Lv et al. found compelling evidence that elevated SUA levels are an independent risk factor for developing T2DM in middle-aged and older individuals, irrespective of other established risk factors, including components of the MetS [12]. Elevated SUA is associated with increased oxidative stress [10,13], which may be a factor in the development of T2DM. The correlation between diabetic neuropathy and hyperuricemia was studied by Yu et al. Further studies should be performed to confirm this hypothesis, but peripheral neuropathy was more common in patients with higher SUA [14].

### 2.5. Neurological Effect

Recognition of higher SUA as a beneficial neurological factor has been a matter of debate. Several studies suggest that lower SUA might be related to the presence of multiple sclerosis [15], Parkinson or Alzheimer’s disease [16], although others disagree with this view about the supposed protective effect of UA on neurological diseases [17]. It is definitely not possible to clearly define the function of SUA on the neurological system.

### 2.6. Cardiovascular Risk

#### 2.6.1. Uric Acid in Cardiovascular Pathology

Several hypothetical mechanisms contribute to the possible cardiovascular risk caused by UA and play a role in the damage of the cardiovascular system. It should be noted that high SUA is associated with metabolic, renal, diabetic, and liver damage in metabolic dysfunction-associated fatty liver disease (MAFLD) and obesity, all of which are predisposing factors for cardiovascular diseases (CVD). An important action to consider is that UA is an outcome of xanthine oxidoreductase (XOR) activity, which is a significant contributor to ROS and oxidative stress. Additionally, XOR is closely connected to nicotinamide adenine dinucleotide phosphate (NADPH) oxidase, which also produces ROS and, as a result, contributes to a higher risk of heart diseases [18]. The presence of UA and XOR has been detected in atherosclerotic plaques, indicating their involvement in the development of atherosclerosis [19]. This may affect the cardiovascular risk in patients with current ischemic heart disease. Although UA functions as an antioxidant and is crucial for protection against oxidative stress, in certain environments, such as the cytoplasm or atherosclerotic plaques, it can convert into a pro-oxidant. UA can also oxidize partially oxidized low-density lipoproteins (LDL), leading to further oxidation. The reduced availability of nitric oxide (NO) is a major and widely accepted pathophysiological mechanism through which SUA promotes CVD. SUA reacting rapidly with NO reduces its availability, converting NO into 6-aminouracil. This results in the absence of an essential chemical compound necessary for proper endothelial function. Atherosclerotic changes are also caused by the activation of cytokines interleukin-1β (IL-1β), IL-6, and tumor necrosis factor-α (TNF-α), which are produced by mononuclear cells stimulated by UA. This shows that SUA has a proinflammatory effect [20].

#### 2.6.2. Coronary Heart Disease, Stroke or Mortality

The correlation between coronary heart disease (CHD), stroke, or morbidity and SUA levels is unclear, and the results of studies on different populations are inconsistent. Two large studies in Asian populations have reported significant associations between SUA levels and CHD or mortality, and the correlation between ischemic heart disease (IHD) and UA was proven [21,22]. The meta-analysis by Li et al. also confirmed the correlation between hyperuricemia and the heightened risk of CHD. Hyperuricemia was likely to elevate the risk of CHD occurrences, especially mortality related to CHD in females [23]. However, many studies concluded that the role of UA in predicting CHD-related deaths is still uncertain and found no significant association between SUA and CHD [24,25,26]. Moreover, in the ALL-HEART trial, which was a large, randomized study of patients with CHD and no history of gout, allopurinol did not improve major cardiovascular outcomes [27]. Thus, due to methodological reasons, it is impossible to establish any conclusion on active decreasing levels of SUA and clinical outcomes based on the ALL-HEART. In general, it remains uncertain whether the associations between UA and mortality vary based on gender, race, or baseline cardiovascular risk and also if it is an epiphenomenon or a risk factor. The extent to which traditional risk factors modify the link between UA and cardiovascular mortality has thus far not been thoroughly investigated.

#### 2.6.3. Congestive Heart Failure

Observational studies focusing on pathophysiology have demonstrated that UA is a reliable marker for the aerobic metabolic capacity in heart failure (HF), and can predict impaired oxidative metabolism, anaerobic threshold, and inefficiency in glucose metabolism [28,29]. A correlation between congestive heart failure (CHF) and SUA has been extensively investigated. In patients with CHF, high levels of SUA are often observed. Many studies have been conducted that investigate the relationship between UA and the incidence, severity, or prognosis of CHF. A meta-analysis performed by Huang et al. showed that having high levels of SUA is linked to a significantly higher risk of developing HF, and the risk increases with higher levels of UA. Elevated SUA levels are also predictive of increased risk for all-cause mortality, cardiovascular mortality, and a combination of death or cardiac events in patients with HF [30]. A study that determined the impact of SUA on CHF concluded that elevated SUA levels are associated with diastolic dysfunction in CHF; however, there was no correlation between UA levels and indicators of left ventricular volumes or systolic function [31]. 

#### 2.6.4. Hypertension

Many studies indicate that hyperuricemia is associated with a higher relative risk of developing hypertension over five years, even when other risk factors are taken into account [32]. The noninflammatory activation of the renin–angiotensin system (RAS) and inhibition of nitric oxide synthase in the kidney, along with stimulation of NADPH oxidases, resulting in mitochondrial dysfunction and increased oxidative stress, is the most well-established mechanism of urate-induced hypertension [7]. Altogether, this promotes endothelial dysfunction, the proliferation of vascular smooth muscle cells, and the reabsorption of sodium, which results in hypertension. Activation of the immune system can be caused by both UA and molecules released in response to the hypertension-induced damage. UA can cause inflammation by engaging nucleotide-binding oligomerization domain and leucine-rich repeat-containing proteins (NLRP3) inflammasome and inducing the cleavage of proinflammatory cytokines IL-1β, IL-6, and TNF-α. Toll-like receptors are also activated and the activation leads to the promotion of inflammatory responses. Additionally, UA can stimulate B-cells which contribute to endothelial impairment and vascular remodeling. Whether a lowering SUA level treatment is beneficial for patients with hypertension is still uncertain and requires thorough investigation in a bigger population [33].

## 3. Pleiotropic Effects of SGLT2i

**Figure 2:**
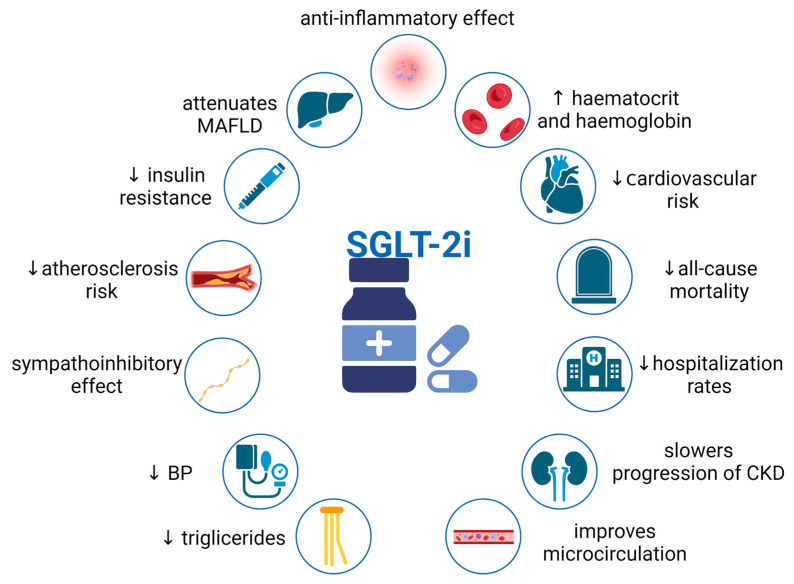
Pleiotropic effects of SGLT2i. Abbreviations: SGLT2i, sodium–glucose cotransporter 2 inhibitors; CKD, chronic kidney disease; BP, blood pressure; MAFLD, metabolic dysfunction-associated fatty liver disease; ↑ increase (level); ↓ decrease (level/risk); created with BioRender.com (accessed on 6 May 2023).

SGLT2is inhibit the sodium-dependent reabsorption of glucose in the kidneys, promoting glucosuria and natriuresis. They were originally used to treat T2DM and have shown significant cardiovascular benefits, which made them a standard treatment in CHF. Clinical trials have shown that SGLT2is can reduce cardiovascular events, all-cause mortality, and reduce hospitalization rates [34]. Additionally, these agents play a role in alleviating further renal damage. Beneficial mechanisms associated with SGLT2is are demonstrated in Figure 2. Kidneys play a crucial role in maintaining glucose and sodium levels by reabsorbing glucose through SGLT transporters in the proximal convoluted tubules of nephrons [35]. SGLT2 cotransporters are primary sodium-dependent glucose transporters responsible for over 90% of renal glucose reabsorption, whereas SGLT1 transport provides less than 10%.

This section may be divided by subheadings. It should provide a concise and precise description of the experimental results and their interpretation, as well as the experimental conclusions that can be drawn.

### 3.1. Renal Effects of SGLT2i—Counteraction against Hyperfiltration, Proteinuria, and Renal Fibrosis

Nephropathy arising from T2DM is associated with increased filtration of a single nephron with normal, increased, or decreased eGFR. The mechanism of enhanced renal perfusion is different than T1DM, involving factors affecting hyperfiltration, such as insulin resistance (IR) or RAS activation. Renal response to SGLT2is in patients with T2DM reduces intraglomerular pressure and single nephron eGFR through vasodilation of efferent arteriole and slight constriction of the afferent arteriole [36]. SGLT2is widely used in T2DM patients might also have an influence on renal protection in T1DM patients. Skrtic et al. revealed the positive effects of SGLTis in T1DM patients with renal hyperfiltration, applying empagliflozin [37]. As a result, renal perfusion was attenuated, probably due to modulation of tubuloglomerular feedback. Whether this therapeutic effect would also apply in T1DM patients with kidney damage is yet to be proven [38]. Another protective effect of SGLT2is is alleviating proteinuria and reducing podocyte remodeling. In a study involving mice with bovine serum albumin-induced proteinuria, dapagliflozin was found to provide a similar level of glomerular protection as an angiotensin-converting enzyme inhibitor (ACE-I), which is the standard comparison treatment [39]. Dapagliflozin effectively reduces proteinuria, glomerular dysfunction, podocyte impairment, and directly targets podocytes by maintaining the architecture of the actin cytoskeleton. Whether SGLT2is could become a potential future treatment option for proteinuria in patients who do not yet have an effective therapy remains to be determined in clinical trials [40,41,42]. It was also reported that SGLT2is could alleviate inflammation and, as a result, renal fibrosis and mesangial expansion [43].

### 3.2. Cardiovascular Effects of SGLT2i

#### 3.2.1. SGLT2i Treatment Decreases Heart Failure Risk

Numerous studies have proven the positive impact of SGLT2is on HF risk. The main result measured in the DAPA-HF study was a combination of either HF deterioration or cardiovascular-associated death. McMurray et al. reported that regardless of T2DM presence in patients, cardiovascular-caused death or worsening of HF was reduced in the dapagliflozin treatment group [44]. Findings established by Packer et al. using empagliflozin were comparable with results of DAPA-HF. Empagliflozin was found to alleviate HF and cardiovascular death risk [45]. Additionally, slower progression of CKD was observed in the study group with HF using empagliflozin. Cardioprotective effect of empagliflozin was also studied by Zhou on diabetic mice [46]. Mice treated with empagliflozin developed smaller zones of hypoperfusion, because of improved microcirculation. Parameters such as endothelial relaxation ability, endothelial nitric oxide synthase (eNOS), phosphorylation, and microvascular density determining microcirculation were evaluated in the myocardium of the mice. Treatment with empagliflozin improved these parameters, and additionally, upgraded the integrity of microvessels, which may lead to a lower chance of experiencing vascular inflammation and microthrombus formation. Repressing excessive mitochondrial fission might play a key role in the beneficial effects on the microvascular endothelium. 

#### 3.2.2. Lipid Profile and Blood Pressure

An additional impact of SGLT2is that deserves attention is their influence on lipid levels. Studies discovered that SGLT2i usage resulted in notable rises in total, LDL, and high-density lipoprotein (HDL) cholesterol levels. However, in patients who received SGLTis, plasma triglyceride levels were lowered. No noteworthy variations in the ratio of LDL to HDL cholesterol were detected as a result of the treatment [47]. Another positive aspect affected by SGLT2is is a significant reduction in blood pressure (BP) [48]. It has been proven that the use of SGLT2is lowers both systolic and diastolic BP in patients with controlled and untreated hypertension.

#### 3.2.3. SGLT2i—Is There Any Influence on the Renin–Angiotensin System?

Considering the fact that SGLT2is promote natriuresis, it is plausible to suggest that they may activate the RAS by lowering levels of sodium. Li et al. aimed to study the effect of SGLT2is on systemic and intrarenal RAS activity [49]. Their data indicated that plasma renin activity (PRA) was enhanced only temporarily and did not affect systemic renin secretion over the long term. There were no changes observed in aldosterone levels after treatment with SGLT2is. The outcomes of another study [50] also indicated that SGLT2is may have an immediate effect on PRA but not plasma aldosterone concentration (PAC). 

Moreover, the long-term activation of PRA and PAC, which could lead to cardiovascular complications, was not detected. However, the intrarenal RAS activity is proven to be changed by SGLT2i use [51].

#### 3.2.4. SGLT2is Change Sympathetic Nervous System Activity

Studies in both humans and laboratory animals suggest that activation of the sympathetic nervous system (SNS) is a key factor in MetS [52,53]. Common strategies for treating MetS, such as weight loss through diet and exercise, aim to suppress sympathetic activity and reduce its influence on hypertension, obesity, and cardiorenal problems [54]. SGLT2is have been found to have sympathoinhibitory effects that could lead to reduction in sympathetic nervous activity (SNA) in the heart and kidneys [55]. Studies have shown that SGLT2is can improve the circadian rhythm of sympathetic activity in rats with MetS and reduce the high-fat-diet-induced elevation of tyrosine hydroxylase and noradrenaline in the kidneys and hearts of mice. This suggests a possible protective effect of SGLT2is through suppressing the renal afferent nerve and alleviating SNA [56,57].

#### 3.2.5. SGLT2is As Myocardium Protectors

Another beneficial effect of SGLT2is is their protection of myocardium. SGLT2is has been shown to modulate myocardial fibrosis by inhibiting the activity of the sodium/hydrogen exchanger 1 (NHE1). This inhibition of NHE1 activity results in a reduction of calcium influx into the myocardium, leading to a decrease in mitochondrial dysfunction. The available evidence suggests that SGLT2is has a protective effect on the myocardium by regulating its metabolism and promoting autophagy in the cells [51]. Another study, performed on diabetic mice, has shown that empagliflozin prevents myocardial fibrosis and might be a therapeutic option for people with diabetic cardiomyopathy [58]. Nevertheless, further clinical trials should be performed to confirm this effect.

### 3.3. Other Effects

Another way that SGLT2is act is their ability to alleviate IR by modifying inflammatory factors induced by T2DM [59]. Okauchi et al. reported that luseogliflozin reduced IR in T2DM mice and had a protective effect on pancreatic β-cells. The administration of luseogliflozin resulted in a significant increase in insulin biosynthesis and secretion, and mRNA expression of insulin was also enhanced [60]. SGLT2is are believed to protect pancreatic β-cells from elevated glucose levels, which can induce β-cell glucose toxicity [61]. Specifically, the activation and recruitment of M2 macrophages is a factor in the development of insulin sensitivity. It also attenuates MAFLD and reduces hepatic inflammation. SGLT2is have been found to have beneficial effects on limiting MAFLD through their direct downregulation of multiple processes, including ROS activity, inflammation, autophagy, or endoplasmic reticulum stress [62,63]. Recent studies suggested that the production of ketones could also alleviate IR and is bound with SGLT2i use [51]. The potential anti-inflammatory effect of ketones was a starting point to research the link between SGLT2is and ketogenesis, which was enhanced in empagliflozin-treated patients. Ferrannini et al. have suggested that hyperketonemia and, specifically, the presence of b-hydroxybutyrate, were correlated with a lower HF risk [64]. Moreover, SGLT2is were associated with a lower atherosclerosis risk due to a decrease in low-grade inflammation by limiting the action of immune cells [65]. It was also proven that SGLT2is can reduce multiple cytokines levels [66]. SGLT2is are also believed to upgrade hematocrit and hemoglobin levels in diabetic patients, which may improve oxygen delivery to tissues in patients with HF [46,51]. The same stimulating mechanism of canagliflozin on erythropoietin production was proven in patients with chronic kidney disease, but further studies should be performed to confirm this effect [67].

## 4. SGLT2i—Potential Mechanism of Serum Uric Acid Reduction

### 4.1. Renal Urate Management

The mechanism of renal urate management is rather complicated, but altering its excretion is mostly achieved by changing its reabsorption and secretion in the proximal convoluted tubule. The main transporters responsible for urate reabsorption are human urate transporter 1 (URAT1) and facilitative glucose transporter 9 (GLUT9). The former and isoform 2 of the latter are responsible for transporting UA through the apical membrane of the cells. Organic anion transporters 4 and 10 (OAT4 and OAT10) also contribute to this effect but to a lesser extent. Isoform 1 of GLUT9 transports UA through the basolateral membranes, thus increasing SUA. Secretion of UA takes place in more distal segment of the nephron. Reuptake of UA from circulation to epithelial cells is conducted by OAT1 and OAT3 and its secretion into the lumen of the nephron takes place through multidrug resistance-associated protein 4 (MRP4) and ATP-binding cassette subfamily G member 2 (ABCG2) [68].

### 4.2. Evidence from Clinical Studies

**Figure 3:**
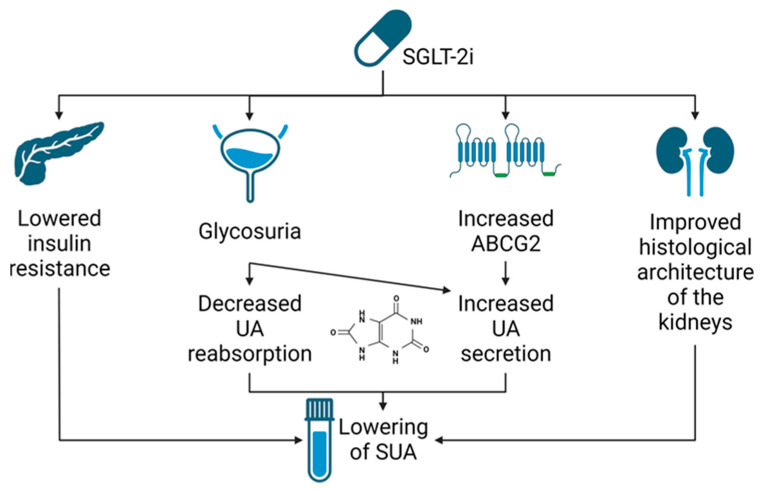
Potential mechanisms of Serum Uric Acid Reduction. Abbreviations: SGLT2i, Sodium–glucose cotransporter 2 inhibitors; SUA, serum uric acid; UA, uric acid; ABCG2, ATP-binding cassette subfamily G member 2. Created with BioRender.com (accessed on 6 May 2023).

The exact mechanism of lowering SUA by SGLT2is remains unknown; however, most authors suggest that it takes place because of the increase in the urinary excretion rate of uric acid (UEUA) [69,70,71,72]. Most of our knowledge on its exact mechanism is based on animal and in vitro studies; however, there were a few attempts to explain this effect clinically. Possible mechanisms of SUA reduction by SGLT2is are illustrated in Figure 3.

An analysis of the first two clinical studies of luseogliflozin showed that SUA was lowered in healthy subjects even after a single dose and a negative correlation between SUA and UEUA occurred. UEUA was also positively correlated with urinary d-glucose excretion and with SGLT2i concentration; however, the first correlation was stronger. The biggest change in SUA in a multiple-dose study was shown on day 1; however, the renal clearance of UA remained and increased for the whole 7 days of the study [69]. A longer observation was conducted on a group of T2DM patients who were administered tofogliflozin. It showed that SUA was lowest after 4 weeks of treatment and then plateaued, while glycated hemoglobin concentration (HbA1c) was lowest after 24 weeks. Patients with lower urinary N-acetyl-β-d-glucosaminidase (NAG)–creatinine ratio, which is a tubular damage marker, responded to treatment better [73]. This, combined with the results from a meta-analysis that showed that the UA lowering effect was decreased in patients with lower eGFR, suggests the importance of kidney function in urate metabolism [74]. Another clinical study involved patients with uncomplicated T1DM. It showed that hyperglycemia increased the fractional excretion of UA and lowered SUA; however, after the introduction of SGLT2is during clamped euglycemia, the same effect was shown, suggesting that glycosuria rather than hyperglycemia was its cause [75]. These studies are consistent in explaining the lowering of SUA by its increased UEUA and associate it with urinary glucose handling; however, to find the exact underlying mechanism, preclinical studies must be taken into consideration.

### 4.3. Evidence from Preclinical Studies

In vitro experiments on cultured cells showed that UA was not transported directly by SGLT2 and revealed no effect of the SGLT2is on the activity of URAT1, GLUT 9 isoform 1, OAT4, OAT10, or sodium-coupled monocarboxylate transporter 1 (SMCT1) [69]. However, injecting cRNA for GLUT9 isoform 2 (which is located in the apical membrane of tubular epithelial cells and transports D-glucose and UA in opposite directions) into Xenopus laevis oocytes increased their efflux rate of UA. What is more, the function of this trans-porter was altered by different concentrations of D-glucose. A concentration of 10 mM, which occurs in the proximal tubule after administration of SGLT2is as an effect of decreased glucose reabsorption, increased excretion of UA and a concentration of 100 mM occurring in collecting ducts inhibited the reabsorption of UA. It showed that even though SGLT2is doesn’t directly alter the activity of UA transporters, glycosuria, which it can cause, has an important influence on them [69].

Studies on animals can also provide some crucial information. Unfortunately, there are some differences between humans and rodents regarding UA handling. The most important one is the lack of uricase in humans, which is present in most other mammals. It breaks down UA to allantoin, which can be excreted in higher concentrations; thus, achieving hyperuricemia is much more difficult in rodents and UA handling may be altered by its decreased concentration [72]. Nevertheless, uricase can be pharmacologically blocked and studies on rodents might bring us some new theories that can be later checked in humans.

First, to support the hypothesis that the main mechanism of lowering UA by SGLT2is is rather by its higher excretion than changes in its metabolism, it is important to show a study on the effect of dapagliflozin on serum XOR activity. It was decreased in fructose-fed rats (an animal model of MetS) but no influence of SGLT2is on its activity was shown [71]. The same study showed increased expression of URAT1, urate transporter (UAT), sodium-dependent phosphate cotransporter 1 (NPT1), and decreased ABCG2 with no changes in GLUT9 or NPT4 in this model. Dapagliflozin decreased the expression of UAT and NPT1 only. Immunohistochemistry and western blot showed an increase in URAT 1, UAT, and GLUT9, which dapagliflozin suppressed; however, only the latter achieved a point of no statistical importance when compared to control. These changes led to increased fractional excretion of UA and lowering of SUA, once more confirming this hypothesis [71]. A study on the T1DM model in rats after streptozotocin treatment showed that lowered levels of insulin resulted in no changes in NPT1, NPT4, or GLUT9 levels, but the expression of URAT1 increased and expression of ABCG2 was decreased. Thus, the fractional excretion of UA was elevated.

These changes were alleviated by insulin administration, but no change was shown after ipragliflozin. The role of insulin in the change of renal UA handling was confirmed in a rat kidney epithelial cell line (NRK-52E) where URAT1 levels were increased after its administration. The same effect was shown in healthy rats as URAT1 was significantly increased, and ABCG2 decreased after insulin. It resulted in increased SUA as an effect of higher reabsorption. Once more, SGLT2is did not alter the levels of these transporters even though the glycemic control was similar in both probes [76]. It shows that insulin, but not hyperglycemia, might be an important factor in renal UA handling, which should be taken into further consideration mainly when considering the effects of another cited study [75] in which clamped euglycemia was achieved by insulin administration as it may alter the results. Experiments conducted on T2DM mice with induced hyperuricemia also showed an SUA-lowering effect by increased UEUA after empagliflozin. However, it was achieved by an upregulation of ABCG2 and the AMPK/AKT/CREB pathway was identified as responsible for it. What is more, no effect on OAT1, OAT3, URAT1, or GLUT9 levels was shown after SGLT2is; however, mRNA for URAT1 was significantly reduced in kidneys. The effect of SGLT2is on ABCG2 was also confirmed on human tubular epithelial cells (HK-2) and the same pathway was confirmed by using Compound C (AMPK inhibitor) [70]. Another study on mice showed a couple of interesting relationships. First, both genetic and pharmacological inhibition of SGLT2 increased UEUA, which suggested that no effect of SGLT2is on urate transporters might be necessary for its function. Second, in mice with a whole-body knockout of the SGLT1, glycosuric and uricosuric effect of SGLT2is was increased, suggesting that, indeed, increased glycosuria might be one of the mechanisms explaining SUA lowering. Finally, the absence of tubular GLUT9 and URAT1 increased basal fractional UA excretion; however, an acute increase in it, as a result of canagliflozin, was not shown when the second transporter was knocked out, suggesting that URAT1 might play an important role in SUA lowering by SGLT2is [72].

Not only kidney excretion of UA might be important in explaining the SUA-lowering effect of SGLT2is, as changes in ABCG-2 expression and in AMPK/AKT/CREB pathway were also shown in the ileum in T2DM mice; however, no changes in GLUT9 in the ileum were shown. It shows that the ileum may also play an important role in UA handling. What is more, the histological picture of the ileum and kidney were improved after empagliflozin in mice with increased SUA, which may suggest a novel mechanism of SGLT2i influence on SUA [70]. In conclusion, direct effect of flozines on UA metabolism and direct involvement of SGLT2 in urate transport were excluded. The main proposed mechanism of lowering SUA by SGLT2is suggests that increased UEUA is its main cause, but the exact mechanism remains unknown. There is some evidence of SGLT2i influence on ABCG2 and URAT-1 and GLUT-9 in it; however, the changes in different studies are inconclusive and the lowering of SUA after genetic SGLT2 inhibition showed that it is not necessary for the effect. The best-documented cause of increasing renal excretion of UA is glycosuria, which may be the main underlying abnormality. However, it certainly is not responsible for all of the effect, as changes in serum insulin, the histological picture of kidneys, and ileal transport of UA might also be of some importance.

## 5. SGLT2i—Effect on Serum Uric Acid Concentration: Findings in Clinical Studies

Since 2013, when the first SGLT2i, canagliflozin, was registered, flozins became an object of interest of many scientists. Since then, many clinical trials and observational studies that revealed the effect of SGLT2is on SUA concentration were published. Furthermore, there are several meta-analyses that explored this topic. As meta-analyses and systematic reviews are considered to be the most trustworthy source of evidence and listing all available studies was not feasible, we decided to present findings from nine meta-analyses that combined and reviewed 7 to 62 randomized controlled trials (RCTs) in both diabetic and nondiabetic patients [74,77,78,79,80,81,82,83,84]. Two of them did not consider SUA reduction as a major outcome [77,78].

### 5.1. Effect of SGLT2is on Serum Uric Acid Concentration

All agents within the drug class exhibited a significant effect on SUA reduction (Table 1). SGLT2is available in Europe and in North America that were examined in the abovementioned meta-analyses—empagliflozin, dapagliflozin, and canagliflozin—reduced SUA concentration by 35.19–45.83 µmol/L, 30.32–41.50 µmol/L, and 36.27–41.22 µmol/L, respectively. It was observed not only in phase 4 cardiovascular outcome trials [34], but also in real-world studies [85]. The hypouricemic effect was detected already in the first weeks of SGLT2i use [86,87,88] and it was sustained throughout a long period of time [34,74,89]. Zanchi et al. also confirmed this impact at 1 month but did not find any acute change in SUA levels 180 min after administration [90]. Additionally, SGLT2is reduced SUA concentration both in young and in elderly patients [91,92]. Furthermore, in the EMPEROR-reduced trial, no significant difference in terms of lowering SUA levels between men and women was observed [93].

### 5.2. Drug- and Dose-Dependency

The findings regarding the association between the SUA-lowering effect and specific agents are inconsistent. While Xin et al. suggested that there was no significant difference between SGLT2is [79], there were some studies that pointed out the supremacy of several SGLT2is. Both Yip et al. and Hu et al. considered luseogliflozin to be more effective in lowering SUA concentration than other agents [82,84]. In contrast, Akbari et al. suggested that empagliflozin had the highest mean SUA reduction among administered drugs [83]. Similar results were seen in a study comparing empagliflozin to dapagliflozin [94]. Many theories could provide background for potential drug-to-drug differences in SUA-lowering effects between flozins. According to the authors of this review, it is notable that empagliflozin and luseogliflozin, considered the strongest for this particular effect, have the highest SGLT2/SGLT1 selectivity (2500 and 1800, respectively). It is much higher than those indices for dapagliflozin (1200) or canagliflozin (200) [95,96]. However, it demands further studies to connect SGLT2/SGLT1 selectivity with SUA-lowering effects. Similar inconclusiveness applied to dose-dependency. Although Zhao et al. observed an association between dosage and SUA-lowering effects for dapagliflozin [74], and Hu et al. suggested a similar relationship for luseogliflozin [82], it was not supported in other studies [79,83,84]. Whether there is a most-effective drug and dose regarding SUA reduction or not needs to be further evaluated.

### 5.3. SUA Reduction in Chronic Kidney Disease Patients

There were few studies in which SUA reduction was not found. It was associated mainly with CKD in T2D patients, and the beneficial effect was disappearing at an eGFR of 60 mL/min/1.73m^2^ [74,84,97,98]. Akbari et al. noted that after removing studies that were conducted only in CKD patients, the mean difference change in SUA concentration increased from −35.17 µmol/L to −36.29 µmol/L, from −36.27 µmol/L to −37.44 µmol/L, and from −40.98 µmol/L to −43.79 µmol/L for dapagliflozin, canagliflozin and empagliflozin respectively [83]. It was also observed that in patients who did not benefit in terms of SUA reduction, eGFR was significantly reduced 12 weeks after introducing treatment with luseogliflozin [87]. On the other hand, in the EMPEROR-reduced trial, empagliflozin seemed to lower SUA concentration consistently across the subgroups regardless of kidney function, even in patients with eGFR lower than 30 mL/min/1.73 m^2^ [88]. It is essential, that while SGLT2is may not significantly reduce SUA in patients with impaired kidney function, regarding their positive effect and improvement in terms of renal and cardiovascular outcomes, they are indicated in CKD patients regardless of diabetic status [99,100].

### 5.4. Factors Influencing SGLT2i Efficacy in Reducing Serum Uric Acid Level

Among the factors influencing the efficacy of SGLT2is in reducing SUA levels were HbA1c, the presence and duration of diabetes, baseline SUA level, and, as mentioned before, eGFR. It was established that lower levels of HbA1c enhanced the hypouricemic effect of SGLT2is [74,85,87]. Baseline SUA concentration was positively associated with its greater reduction [73,85,87,88], but it did not influence the beneficial effect of flozins on cardiovascular outcomes [88,89]. Moreover, Ouchi et al. suggested that a greater decrease in HbA1c was observed in patients with higher baseline SUA levels [73]. The EMPEROR-reduced trial and the DAPA-HF showed a greater mean SUA reduction in nondiabetic patients [88,89]. Yip et al. performed the only meta-analyses that included both patients with and without diabetes and proved that mean SUA reduction was smaller in diabetic patients than in nondiabetic patients, 31.48 µmol/L and 91.38 µmol/L, respectively [84]. There was a lack of consistency regarding the duration of diabetes. Whereas some studies suggested a negative association between longer duration of the disease and SGLT2is ability to reduce SUA [74], other research indicated the opposite relationship [83].

### 5.5. Effect of SGLT2is on Acute Gout Events and New Antigout Drugs Commencement

Besides exploring the direct effect of SGLT2is on SUA concentration, several studies were conducted that examined the association between SGLT2i use and commencement of new antigout drugs and incident gout events [89,101,102]. Banerjee et al. conducted a meta-analysis that reviewed and combined five studies, noting the scarcity of literature on experimental and observational studies [103]. Compared to placebo, SGLT2is significantly reduced both the new antihyperuricemic drugs commencement (pooled HR 0.58) and composite gout outcomes (pooled HR 0.61) in diabetic patients. A similar association was observed comparing SGLT2is to other oral glucose-lowering drugs, such as glucagon-like peptide-1 (GLP-1) agonists and dipeptidyl peptidase 4 inhibitors (DPP4i) [103].

### 5.6. SLGT2i—Novel Mechanism of Action

Hyperuricemia and acute gout events are associated with increased risk of major adverse clinical outcomes, such as hospitalization for HF or cardiovascular mortality [88]. As medication administered during acute gout flare-ups, such as nonsteroidal anti-inflammatory drugs or steroids, are very often contradicted in HF or CKD patients, the new, safe drug is in demand. In the QUARTZ study, dapagliflozin was administered in subjects with asymptomatic hyperuricemia as a third drug in combination with antigout drugs, verinurad and febuxostat [104]. It was observed that dapagliflozin further reduced SUA, with no adverse outcome regarding kidney function. There was concern that a further increase in UEUA may lead to crystallization in renal tubules and, eventually, nephrolithiasis. However, this concern was not supported by available evidence [105].

## 6. Conclusions

Increased SUA may be a cause of many different diseases, starting from gout, in which it is a direct cause of ailments, through T2DM, hypertension, and CHF, in which hyperuricemia is a proven risk factor, to CHD, in which the correlation remains unclear. What is more, increased SUA may be a protective factor against developing multiple sclerosis, Parkinson’s, and Alzheimer’s diseases; however, there is no agreement between authors on this matter. SGLT2is have a diverse impact on the human body. They have some influence on RAS, SNS activity, lipid profile, BP, atherosclerosis risk, hematocrit, and hemoglobin. They can also alleviate IR by modifying inflammatory factors and renal damage by counteracting hyperfiltration, proteinuria, and renal fibrosis. What is more, they reduce cardiovascular events, all-cause mortality, and hospitalization rates in some groups of patients. Some of these effects may be due to their impact on SUA. All of the cited meta-analyses showed that, indeed, SGLT2is significantly reduce SUA

The data on the drug- and dose-dependency of this effect are inconclusive, similar to the influence of diabetes duration and eGFR; however, some alternating factors such as HbA1c, presence of diabetes, and baseline SUA level were broadly accepted. Even though there is a consensus that the lowering of SUA by SGLT2is is due to increased UEUA rather than its altered metabolism, its exact mechanism remains unknown. Many possible pathways were proposed, and they all may have some influence. Genetic inhibition of SGLT2 showed an SUA-lowering effect, which suggested that direct influence on UA transporters is not necessary; however, other studies showed many differences in their expression and activity after SGLT2i. The influence of SGLT2is on SUA may not only be used in gout treatment but may also be of huge importance in explaining the observed pleiotropic effects of SGLT2i. Further studies to assess this effect of SGLT2is are necessary to explore its exact mechanism and to fully utilize the potential of SGLT2i.

## Figures and Tables

**Table 1:** Effects of SGLT2is on SUA concentration—summary of meta-analyses.

Author	Year	No. of Trials	No. of Individuals	Population	Drug	Uric Acid (µmol/L)	I^2^
Musso [77]	2012	7	2943	T2D	dapagliflozin	−41.50 (−47.22, −35.79) ^a^	50%
Zhang [78]	2014	10	3464	T2D	dapagliflozin	−36.17 (−40.99, −31.36) ^a^	64%
Zhao [74]	2018	62	34,941	T2D	total	−37.73 (−40.51, −34.95) ^a^	73.5%
					empagliflozin	−45.83 (−53.03, −38.63) ^a^	0%
					dapagliflozin	−36.99 (−41.73, −32.25) ^a^	65.4%
					canagliflozin	−41.22 (−45.03, −37.42) ^a^	81.4%
					ipragliflozin	−17.40 (−23.78, −11.02) ^a^	12.5%
					luseogliflozin	−28.20 (−34.73, −21.67) ^a^	11.6%
					tofogliflozin	−21.48 (−35.15, −7.81) ^a^	0%
Xin [79]	2019	31	13,650	T2D	canagliflozin	−37.02 (38.41, −35.63) ^a^	80%
					dapagliflozin	−38.05 (−44.47, −31.62) ^a^	99%
					empagliflozin	−42.07 (−46.27, −37.86) ^a^	67%
					tofogliflozin	−18.97 (−28.79, −9.16) ^a^	7%
					ipragliflozin	−19.75 (−28.17, −11.34) ^a^	0%
Zhao [80]	2019	12	5781	T2D	Empagliflozin ^c^	−36.59 (−46.22, −26.96) ^a^	65%
					Empagliflozin ^d^	−43.55 (−52.40, −34.70) ^a^	65%
Wu [81]	2019	10	5159	T2D	total	−26.16 (−42.14, −10.17) ^a^	80%
Hu [82]	2022	19	4218	T2D	total	−0.965 (−1.029, −0.901) ^a,b^	98.7%
					empagliflozin	−0.710 (−0.832, −0.587) ^a,b^	0%
					dapagliflozin	−2.787 (−2.965, −2.610) ^a,b^	98.9%
					canagliflozin	−0.503 (−0.639, −0.366) ^a,b^	0%
					ipragliflozin	−0.294 (−0.438, −0.151) ^a,b^	87.6%
					luseogliflozin	−6.916 (−7.288, −6.544) ^a,b^	97.9%
					tofogliflozin	−0.184 (−0.357, −0.011) ^a,b^	86.3%
Akbari [83]	2022	55	36,215	T2D	total	−34.07 (−37.00, −31.14) ^a^	78.8%
					empagliflozin	−40.98 (−47.63, −34.32) ^a^	84.9%
					dapagliflozin	−35.17 (−39.68, −30.66) ^a^	73.9%
					canagliflozin	−36.27 (−41.62, −30.93) ^a^	66.5%
					luseogliflozin	−24.269 (−33.31, −15.22) ^a^	66.3%
					tofogliflozin	−19.47 (−27.40, −11.55) ^a^	0%
					ipragliflozin	−18.85 (−27.20, −10.49) ^a^	59%
Yip [84]	2022	43	31,921	total	total	−33.03 (−37.38, −28.69) ^a^	92%
		3	597		luseogliflozin	−47.73 (−79.50, −15.96) ^a^	94%
		7	4002		canagliflozin	−36.62 (−42.67, −30.56) ^a^	61%
		16	17,653		empagliflozin	−35.19 (−42.61, −27.78) ^a^	96%
		15	5036		dapagliflozin	−30.32 (−36.20, −24.43) ^a^	67%
		2	702		ipragliflozin	−20.37 (−29.17, −11.56) ^a^	72%
		4	198	without T2D	total	−91.38 (−126.53, −56.24) ^a^	80%
		39	31,723	T2D	total	−31.48 (−37.35, −25.60) ^a^	92%
		8		CKD	total	−8.12 (−22.17, 5.94), *p* = 0.26	69%

Data are presented as mean difference and 95% CI, I^2^—heterogeneity. ^a^ *p* < 0.05; ^b^ standard mean difference; ^c^ 10 mg; ^d^ 25 mg Abbreviations: SGLT2i, Sodium–glucose cotransporter 2 inhibitors; SUA, serum uric acid;T2D, type 2 diabetes; CKD, chronic kidney disease.

## Data Availability

Not applicable.
